# Electrochemical CO_2_ Conversion Commercialization Pathways: A Concise Review on Experimental Frontiers and Technoeconomic Analysis

**DOI:** 10.1021/acs.estlett.4c00564

**Published:** 2024-09-17

**Authors:** Bijandra Kumar, Baleeswaraiah Muchharla, Moumita Dikshit, Saudagar Dongare, Kapil Kumar, Burcu Gurkan, Joshua M. Spurgeon

**Affiliations:** † Department of Math. Comp. Science and Eng. Technology, Elizabeth City State University, Elizabeth City, North Carolina 27909 United States; ‡ Laboratory of Environmental Sustainability and Energy Research (LESER), National Institute of Technology Delhi, New Delhi, 110036 India; § Department of Chemical and Biomolecular Engineering, Case Western Reserve University, Cleveland, Ohio 44106 United States; ∥ Conn Center for Renewable Energy Research, University of Louisville, Louisville, Kentucky 40292 United States

**Keywords:** Carbon dioxide, technoeconomic analysis, electrochemical conversion

## Abstract

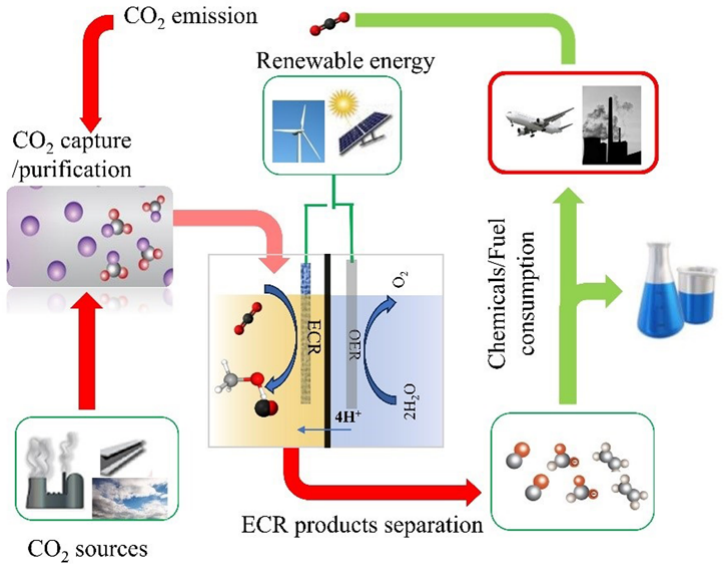

Technoeconomic analysis (TEA) studies are vital for formulating guidelines that drive the commercialization of electrochemical CO_2_ reduction (eCO_2_R) technologies. In this review, we first discuss the progress in the field of eCO_2_R processes by providing current state-of-the-art metrices (e.g., faradic efficiency, current density) based on the recent heterogeneous catalysts’ discovery, electrolytes, electrolyzers configuration, and electrolysis process designs. Next, we assessed the TEA studies for a wide range of eCO_2_R final products, different modes of eCO_2_R systems/processes, and discussed their relative competitiveness with relevant commercial products. Finally, we discuss challenges and future directions essential for eCO_2_R commercialization by linking suggestions from TEA studies. We believe that this review will catalyze innovation in formulating advanced eCO_2_R strategies to meet the TEA benchmarks for the conversion of CO_2_ into valuable chemicals at the industrial scale.

## 1 Introduction

**Figure 1:**
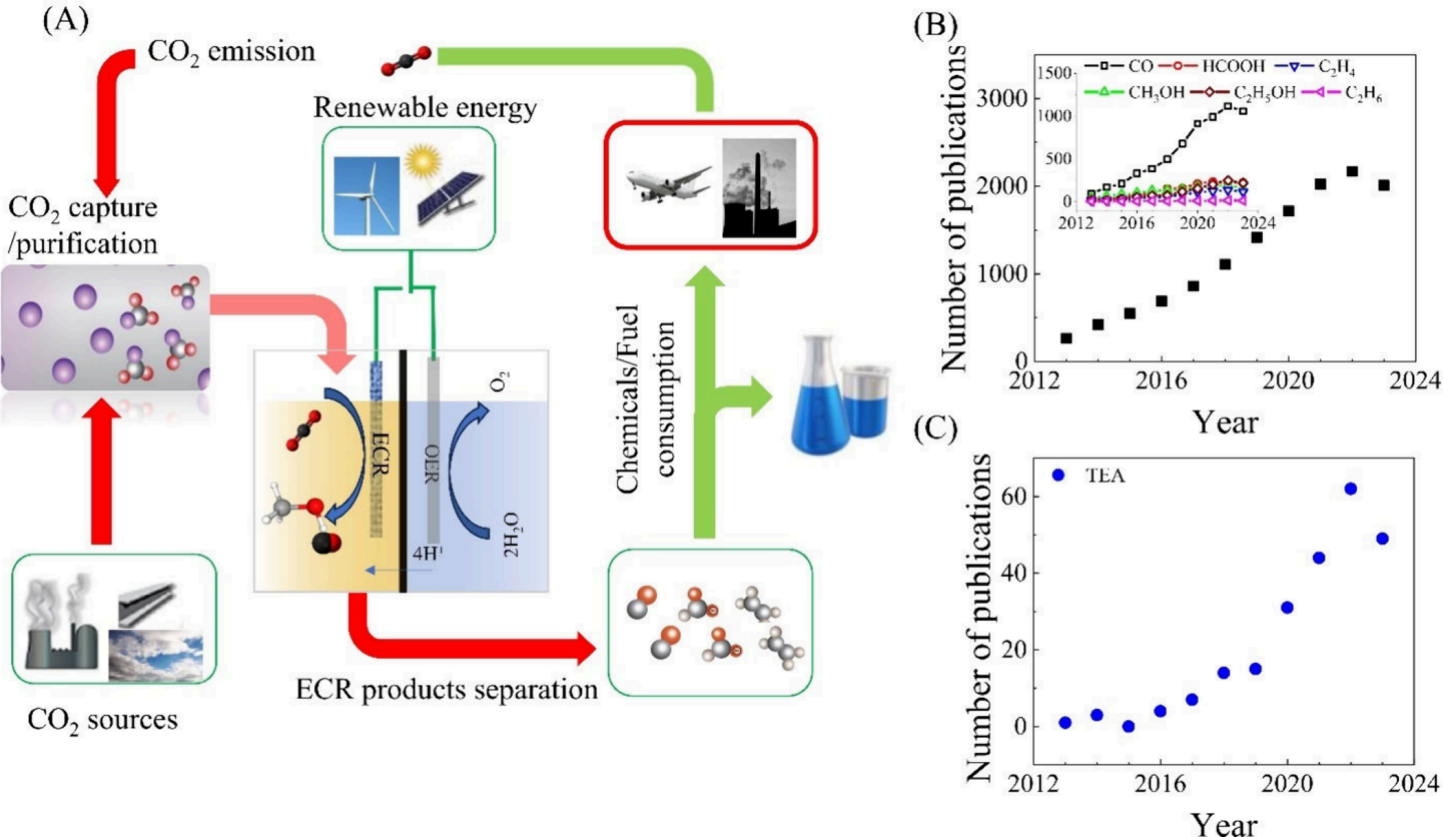
(A) Schematic for CO_2_ recycling by eCO_2_R. Number of publications from Web of Science using the query: “electrochemical” AND “reduction OR conversion” AND “CO_2_ OR carbon dioxide”. (B) Total number of publications and number of publications for various products (inset) and (C) number of publications related to TEA for CO_2_ electrochemical conversion process.

The emergence of a clean energy economy is disrupting the traditional relationship between fossil fuel consumption and global economic growth. In recent years, there has been substantial growth in renewable energy production, with over 500 gigawatts (GW) of additional renewable generation capacity slated for inclusion in 2023 alone.^1^ Despite this, fossil fuel is contributing the most (∼73%) and continues to lead in fulfilling the energy demand throughout this decade.^1^ The consumption of fossil fuels results in the emission of CO_2_ and the accumulated CO_2_ in the environment reaching 36.1 ± 0.3 Gt in 2022.^3^ Here, it should be noted that excessive CO_2_ is considered to be the primary reason for global warming. Hence, significant efforts are directed toward developing a carbon-neutral or carbon-negative economy. In this aspect, a complex approach where different pathways, e.g., renewable energy production, CO_2_ capture and sequestration (CCS), CO_2_ capture and utilization (CCU), and others, are effectively employed analogously to accomplish the goal defined by global policymakers.^4−7^ In terms of CCS, the CO_2_ can be directly used for various industries (e.g., enhanced oil recovery and gas recovery, and enhanced geothermal systems) at large scale.^8^ Alternatively, CO_2_ can be used as a feedstock for producing chemical precursors (e.g., ethylene) and energy dense fuels (e.g., ethanol).^9−13^ Among different CO_2_ conversion processes, the electrochemical CO_2_ reduction (eCO_2_R), preferably integrated with renewable energy sources, offers a scalable and carbon-neutral pathway where CO_2_ captured from the industries (e.g., cement and steel industries) or air can directly be converted into chemical precursors nurturing sustainable synthesis, or energy-dense fuels under mild conditions (Figure 1A).^14−16^ The eCO_2_R process also has advantages over other competitive CCU methods (e.g., photocatalysis, photoelectric catalysis, and biocatalysis) and CSU technologies as the eCO_2_R process is faster, economically feasible, scalable and helps in partially mitigating intermittency via storing renewable energy in the chemical bonds.^10−12,17^

The main challenge with the eCO_2_R is that CO_2_ is notoriously a very stable molecule, thus requiring special catalysts to activate and further reduce it into valuable products.^18^ Once activated, CO_2_ can be reduced to distinct products (carbon monoxide (CO), formic acid (HCOOH), formaldehyde (HCHO), methane (CH_4_), methanol (CH_3_OH), oxalic acid (C_2_H_2_O_4_), ethane (C_2_H_6_), ethylene (C_2_H_4_), ethanol (C_2_H_5_OH), acetic acid (CH_3_COOH) and n-propanol (C_3_H_7_OH)) based on the number of protons coupled with electrons transferred during the eCO_2_R process, adjoining nonelectrochemical steps. Further, there are fundamental challenges related to the selectivity, reaction rate, energy efficiency, durability, competency with incidental hydrogen evolution reaction (HER), and others.^19^ Therefore, significant efforts are directed toward solving these issues. Data analysis collected from the Web of Science suggests that substantial efforts have been dedicated to the eCO_2_R process at the lab scale. These efforts include designing new catalysts,^20−24^ understanding the eCO_2_R mechanism,^25,26^ enhancing product selectivity,^27,28^ defining electrolyzer configurations,^19,29−31^ and exploring new eCO_2_R approaches,^13,32−34^ as evidenced by exponential growth in the number of publications during the past decade (Figure 1B). The findings, described in the inset of Figure 1B, illustrate the annual publication count for distinct products, such as CO (2e^–^), HCOO^–^/HCOOH (2e^–^), CH_3_OH (8e^–^), C_2_H_5_OH (12e^–^), C_2_H_4_ (12e^–^), and C_2_H_6_ (14e^–^).

In terms of commercialization prospects, TEA and life-cycle assessment (LCA) are essential tools that provide input for addressing challenges associated with the commercialization of the eCO_2_R technology. Parameters that factor in include operation costs, the commercial feasibility of different eCO_2_R products, sensitivity of different eCO_2_R metrics, etc., and assessment of its environmental and economic feasibility to determine if the overall process is carbon-negative and commercially profitable. According to recent TEA studies, the formation of CO and HCOOH has been considered more profitable, as the production rate is competitive with commercially available HCOOH and CO produced at industrial scales.^35−37^ Additionally, CO and HCOOH formation is easier as they involve less number of electron transfer reactions compared to other eCO_2_R products; thus, most research efforts have been dedicated to CO and HCOOH. However, multicarbon eCO_2_R products can be more valuable due to their high market value and low cost/electron/molecule production, albeit challenging due to the multistep electron-coupled proton transfer process. Recent efforts are emerging to perform TEA studies to provide guidelines for the scientific community and policymakers for the commercialization of disruptive eCO_2_R technology, as evidenced by the increasing number of scientific publications in recent years. (Figure 1C).

Many reviews have discussed the progress in the field of different type of catalysts (e.g., nanoelectrocatalysts,^38^ bimetallic metal–organic framework electrocatalysts,^39^), different products (e.g., CO_2_-to-CO conversion,^40^ CO_2_-to-alcohol,^14^ CO_2_-to-HCOO^–^/HCOOH,^41^ multiscale CO_2_ electrocatalysis to C_2+_ product^42^), long-term-stability of different catalysts,^43^ durability of electrolyzers or catalysts,^44^ CO_2_ electrolyzers,^31^ importance of catalyst–electrolyte interface,^45^ mechanistic electrocatalysis models and system design,^46^ and scalable carbon dioxide electrolysis.^47^ However, a comprehensive review that discusses the relationship between current state-of-the-art eCO_2_R findings and the TEA study is currently scarce. In this review, we bridge this gap by providing key guidelines that are essential for research in the field of the eCO_2_R process. We also model TEA strategies to offer direction for eCO_2_R research, propelling this disruptive technology toward a more realistic and affordable future. By establishing a connection between cutting-edge eCO_2_R advancements and their technoeconomic feasibility, our review aims to guide researchers, policymakers, and industry stakeholders in shaping the future of sustainable energy solutions.

## 2 Common Metrics for eCO_2_R Process Impacting TEA Study

There are numerous metrics that play a significant role in defining the possibility of an eCO_2_R process for commercialization and the competitiveness of eCO_2_R products with their counterpart products. The key metrices are as follows:

### 2A Faradaic Efficiency (FE)

FE represents how specifically the electrons are transferred in an electrochemical process for targeted product formation. It is the ratio of the charge used to produce a desired product to the total charge passed during the eCO_2_R process. The FE directly and inversely proportional to the energy efficiency (EE) and electrode area of the electrolyzers (eqs 1 and 2).^48^ In terms of the TEA perspective, a higher FE is favorable to minimize the capital and operating cost of the system. In general, the FE for most of the products should be higher than 80%.
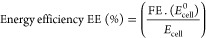
1

Where *E*_cell_^0^ is the thermodynamic reaction potential for particular product and *E*_cell_ is the operating cell voltage. The total energy efficiency of an electrolyzer will be sum of energy efficiencies for all products.^37,49,50^

### 2B Current Density

Current density is defined as the total current flow normalized by the electrode surface or electrochemically active area. The partial current density for any eCO_2_R product represents the fraction of the total current density specifically used for the formation of that particular product. The partial current density is calculated by multiplying FE and the total current density of the reaction. High partial current density is essential for a higher production rate, resulting in the requirement of a smaller electrode area of the CO_2_ electrolyzers (eq 2) and thus lower capital costs.
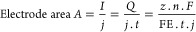
2

Where, *I* is the current, *j* is the current density, *Q* is the total charge in Coulombs, *t* is the operating time, *z* is the number of required electrons to produce the product, *n* is the number of moles of the given product, FE is faradaic efficiency, and *F* is Faraday’s constant (96485 C·mol^–1^).^48^

### 2C Cell Voltage

The cell voltage (*E*_cell_) is an important metric for TEA studies, as the cost related to power consumption is directly related to the energy requirement in electrical equivalents to drive the reaction. The power consumption is directly proportional to the cell voltage (eq 3).^51^
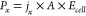
3where *P*_*x*_ is the power required to produce component x, *j*_*x*_ is the partial current density for component *x*, *A* is the electrode area, and *E*_cell_ is the cell voltage. The total required power will be the sum of powers for all products. The cell voltage is the total applied voltage to a CO_2_ electrolyzer. The cell voltage will be minimum if the overpotential and ohmic voltage drop due to electrolyzer configuration-originating resistance in the circuit are minimized.

### 2D Single-Pass Carbon Efficiency (SPCE)

SPCE is the ratio of the CO_2_ concentration measured before and after the eCO_2_R process. This is an important matrix for the commercialization of the eCO_2_R process, directly impacting the overall cost of the eCO_2_R process and commonly ignored in experiments at the lab scale. A greater than 50% SPCE is recommended for the commercialization of the eCO_2_R process.^15^

### 2E Stability

The stability of the catalysts and electrolyzers significantly affects the overall cost of the CO_2_-to-product system. The degradation of the system should be less than 10 μV h^–1^ or higher than 10,000 h.^15,52^ A lower catalyst stability requires more frequent capital expense replacement costs during the plant lifetime of the TEA.

## 3 Progress and Current State-of-the-Art Metrics for Different eCO_2_R Products

The significant efforts made in the last 50 years have resulted in disruptive progress in the eCO_2_R processes. Here, it should be noted that the key metrics discussed above highly depend on the intrinsic properties of the catalysts, type of the electrolyzers, and supportive components such as electrolytes, pH, membrane, and others.^53^

### 3A Catalysts Discovery

In the past decade, historic progress has been seen in electrocatalysts for the eCO_2_R process. Heterogeneous catalysts in diverse forms (e.g., thin film, nanoparticles, nanocubes, nanowire, nanotubes, nanoporous thin film, nanorods, nanoflowers, carbon-based materials, 2D nano materials, reduced metal oxides, organic metal framework, MXene) have been extensively studied for either understanding the fundamental eCO_2_R mechanism or enhancing the eCO_2_R rate, stability, product selectivity.^14,20−22,25,28,36,46,54−65^ Intrinsic properties such as modified electronic structure via doping, nano- and mesoscale effect, subsurface oxygen, pore size and shape, roughness, grain-boundaries play an important role in governing the catalytic activities of the materials.^54,55,66−72^ Recently tandem atomic structures and dual-cathode have led to higher FE for C_2+_ eCO_2_R products via combining CO_2_-to-CO and CO-to-C_2+_ eCO_2_R steps in one system.^73−77^ For example, AgCu tandem catalysts enable producing total C_2_ products with 82.6% FE and C_2_H_5_OH with FE 51.8% at −1.0 V vs RHE, which is significantly higher in comparison to total C_2_ products FE (∼57%) and CO-to-C_2_H_5_OH production FE (∼43%) attained by state-of-the-art oxide derived Cu catalysts.^78,79^ The SnS_2_ nanosheets over 3D carbon have shown remarkable high CO_2_-to-C_2_H_5_OH FE (82.5% at −0.9 V vs RHE) due to a tandem effect where Sn atoms surrounded by three oxygen atoms enhance C–C coupling via a formyle-bicarbonate coupling mechanism.^80^ In another effort, coelectrodeposited CuAg alloy enabled the production of 2-propanol with 56.7% FE and a specific current density of 59.3 mA cm^–2^.^81^ The selectivity for 2-propanol over 1-propanol was attributed to the modified surface binding energy of the intermediates, promoting C–O bond formation. Moreover, the C–C coupling for higher carbon products can further be enhanced by engineering the catalyst surface, surface functionalization or joint tuning of nanostructured catalysts and local electrolyte interface.^23,68,70,82−84^

### 3B Electrolyzers

The fundamental eCO_2_R research has been mostly performed in standard H-type electrochemical cells without a membrane or with a cation exchange membrane (CEM)/anion-exchange membrane (AEM) configuration due to their easier operation, low cost, and requirement of minimal usage of catalysts and electrolyte. It is also feasible to connect H-cell with advanced characterization techniques (e.g., XRD, Fourier Transform Infrared (FTIR), mass spectroscopy and others) for operando characterization tools.^85−87^ For instance, in situ FTIR spectrometry experiments performed during eCO_2_R in acetonitrile solvent revealed that CO_2_ molecules in acetonitrile repel water molecules from the interface, thus simultaneously enhancing the eCO_2_R and strongly inhibiting HER.^86^ For commercial applications, liquid phase (with AEM, CEM and without membrane), gas phase (with AEM, CEM, or bipolar membrane (BPM)), and solid oxide electrolyzers have been designed.^1,46,88−90^ Each type of electrolyzer has its own advantages and limitations.^90^ For example, solid oxide electrolyzers can be operated at high current density (>1 A cm^–2^ and energy efficiency but mostly produced single carbon product.^91,92^ On the other hand, gas-phase electrolyzers can potentially work sufficiently at high current density (>hundreds of mA cm^–2^) but present limitations due to the acidification of cathode, hydration of membrane, increased ohmic losses across the membrane among others. Further effort has been made toward studying eCO_2_R in single chamber electrolyzer (SC electrolyzer) and electro-stacks of electrolyzers.^29,85,93−96^ The configuration and stacking of electrolyzers (parallel or horizontal) have significant advantages over the SC electrolyzer for CO_2_-to-product formation due to higher stability in terms of maintaining operating voltage and current density, low operating cost, high selectivity, and high single CO_2_ pass ratio.

### 3C Electrolyte

The electrolyte is an essential component for the eCO_2_R process since it also serves as the catalyst in the heterogeneous reactions involved. The performance of the eCO_2_R process is subjected to the chemical and physical properties of the electrolytes.^13,88,97−100^ For example, the conventional aqueous electrolytes (e.g., aqueous KHCO_3_, KOH, etc.) are inexpensive, easy to use and widely accessible; however, they suffer from low CO_2_ solubility (∼33 mM – aquas electrolyte), low conductivity (e.g., 10 mS cm^–1^ for 0.1 M KHCO_3_), cation/anion mass transport limitation. Further, the electrochemical stability of the aqueous systems promote HER and the undesired carbonate formation.^101,102^ The performance of the eCO_2_R systems highly depends on the solute CO_2_/carbonate/bicarbonate equilibrium and localized pH.^103,104^ The organic electrolytes (protic or aprotic) offer relatively higher CO_2_ solubility, wider operational voltage window, and more diverse eCO_2_R pathways, promoting C–C coupling, despite the use of similar catalysts as with aqueous electrolytes.^100,105−107^ In aprotic solvents, the formation of a CO_2_* radical anion while a proton or an oxygen transfer from the solvent to the intermediate, or intermediate to the solvent occurs leading to diverse eCO_2_R product.^108,109^ Ionic liquids (ILs) consisting of an organic cation and an inorganic/organic anion has attracted great attention as electrolytes due to their chemically tailorable nature, high CO_2_ solubility, low volatility, high ionic conductivity, high thermal conductivity, and cocatalytic characteristics that enhances overall eCO_2_R process.^110−116^ For instance, in the presence of ILs (e.g., EMIM-BF_4_) CO_2_-to-CO conversion occurs at the lowest overpotential (20 mV) as ILs form CO_2_–ILs complex and reduce the energy barrier for CO_2_^•–^ intermediate formation.^22,117−119^ The intrinsic properties of cation and anion also play a significant role in tuning the eCO_2_R at different catalysts surface via controlling the localized environmental (e.g., pH) conditions.^101,115^ A humidified CO_2_ stream has been used in three-compartment or solid oxide electrolyzers to produce mainly HCOOH with high current density (>200 mA cm^–2^) and stability over 1000 h.^19,120,121^ This approach has proven to be beneficial by eliminating costs associated with the downstream product separation and has additionally been examined for multicarbon product formation on Cu catalysts.^121^ The pH, viscosity, nature of electrolytes, flow rates, ionic species and their size play crucial roles in defining the eCO_2_R mechanism and production rate.^100,104,108,109,122−126^

### 3D Electrolysis Process Design

In the quest of high energy efficiency and capability of C_2+_ product formation with high FE, cascade or tandem eCO_2_R processes have been examined. In this process, two electrolyzers essentially configured to convert CO_2_-to-CO and CO-to-C_2+_ are integrated together.^33,127−130^ The tandem configuration offers freedom of utilizing the most efficient and state-of-the-art two distinct but complementary catalysts/electrolytes, CO_2_-to-CO (e.g., nanostructured Ag/ILs) and CO-to-C_2+_ (e.g., oxide derived Cu/KOH) systems. For example, a CO flow electrolyzer with a well-controlled electrode–electrolyte can achieve a total C_2+_ FE of ∼91% with a C_2+_ partial current density over 630 mA cm^–2^.^131^ The CO can also be reduced to acetate (FE - 48%) with high partial current density (131 mA cm^–2^) using Cu nanosheets.^60^ CO-to-n-propanol has also been selectively produced with a 47 ± 3% FE using Pb–Cu catalysts at high rate (60 mA cm^–2^).^132^ As recapitulated by Overa et al., the higher C_2+_ FE and partial current density can be achieved via CO-to-C_2+_ conversion which is technically and economically challenging by direct CO_2_ electrolysis despite using an advanced catalytic system.^127^ Theaker et al. used nanocoral Ag and oxide derived Cu based catalysts in a tandem systema and able to achieve a FE of 11% for C_2_H_5_OH at an average applied voltage of −0.52 V vs RHE.^33^ However, the system was not optimized as a stream of CO_2_ and CO collected from the first electrolyzer was passed into the second electrolyzers. Cuellar et al., have demonstrated a tandem system consisting of a CO_2_ absorption column between two CO_2_-to-CO and CO-to-C_2+_ converting electrolyzers enable to produce C_2+_ products of 62% at a total current density of −300 mA cm^–2^.^129^

Recently pulse electrolysis has emerged as an alternative approach to tune: (1) the local eCO_2_R environment (e.g., concentrations of reactants, and intermediates); (2) the species within the electrical double layer; (3) the catalyst-adsorbate interactions; and (4) dynamic reorganization/restructuring of catalyst facets.^34,133−139^ Thus, pulsed voltage plays a significant role in eCO_2_R mechanism and kinetics offering essential advantages which are not offered in standard static eCO_2_R process. For instance, the catalyst’s stability can be significantly improved via eliminating salt/impurity-deposition process during prolonged electrochemical reaction without compromising the product selectivity. As an example, in the case of Cu catalysts in an MEA cell, the catalysts performance decreased by 50% within the first 40 h of the test, while FE for C_2_ (∼80%) and eCO_2_R product (∼98%) remain unchanged during 60 s of operation at −3.8 V followed by 30 s of regeneration at −2.0 V pulse for more than 240 operational hours.^136^ Moreover, due to restructuring the Cu surface chemical properties, a tunable CO/H_2_ ration has been obtained as product in place of multiple carbon product in standard experimental conditions.^34^ In another study, Cu has been activated in situ via applying alternating current, resulting in a square-wave alternating reduction and oxidation process. This led to CH_4_ production (FE > 70%) with high yield in the gas product stream (∼23.5%) in contrast to the direct current applications where H_2_ is the main product.^139^

**Figure 2:**
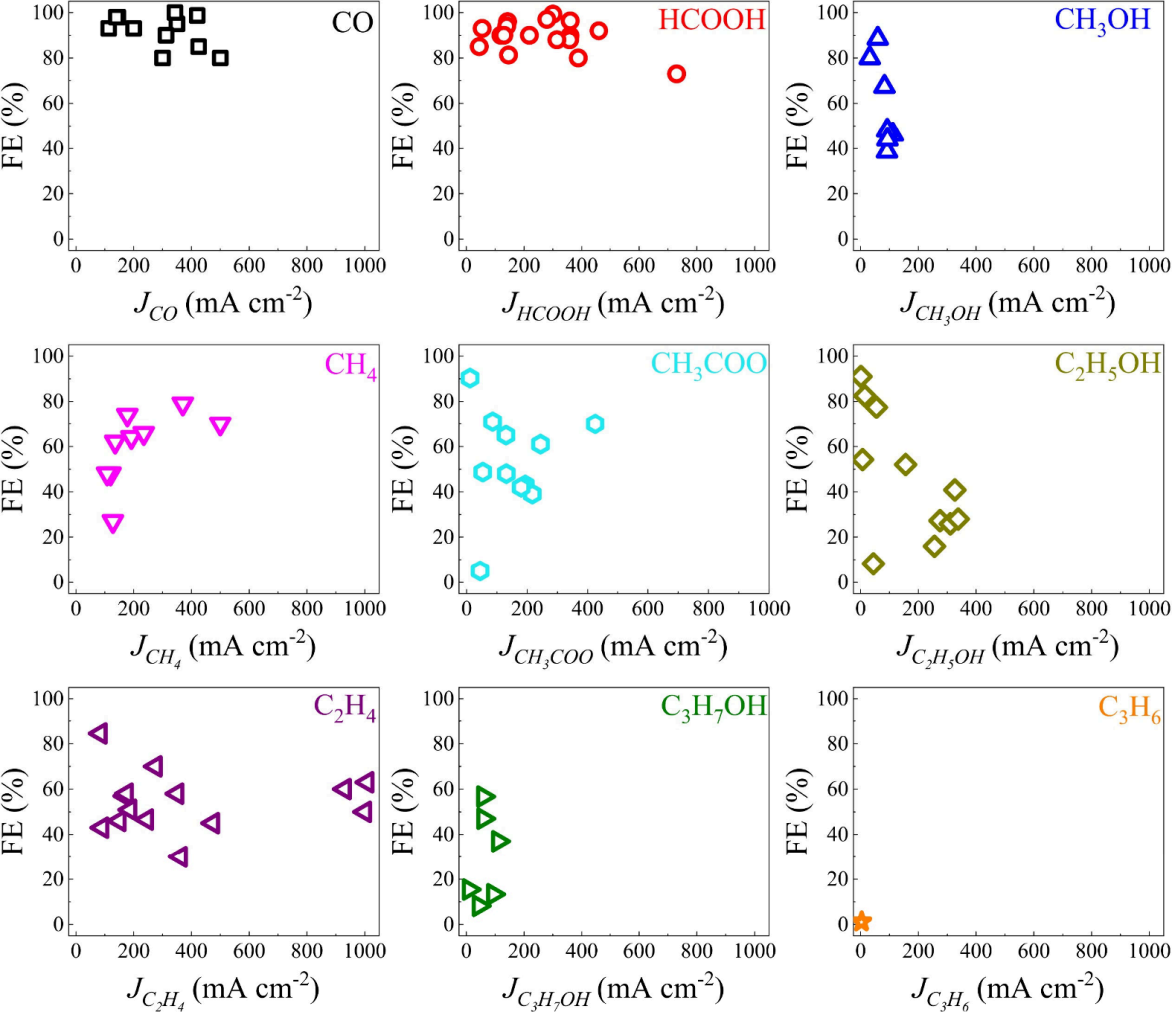
Summary of key metrics (FE and partial current densities) for current state-of-the-art eCO_2_R products across various eCO_2_R systems. The data, meticulously gathered from 81 scholarly publications, have been comprehensively analyzed and summarized in Table S1. It reveals that CO and HCOOH can be produced with higher partial current density and FE. In contrast, the FE and partial current density for eCO_2_R liquid products fall outside the desired range.

To understand the feasibility of eCO_2_R process for commercialization, we summarize the performance of state-of-the-art eCO_2_R systems enable to produce eCO_2_R products with higher current density and FE (Table S1). Recognizing the importance of FE and partial current density in assessing the competitiveness of eCO_2_R products compared to commercially available alternatives, we deliberately plotted FE against partial current density for various eCO_2_R products (Figure 2).^35,37,139,140^ The CO and HCOOH can be produced with higher FE (>80%) and partial current densities (>300 mA cm ^–2^). However, other products (mainly liquid products) required significant progress to meet the industrial goals in terms of current density (>300 mA cm ^–2^) and selectivity (FE > 80%).^15^

## 4 TEA for CO_2_ Electrochemical Conversion Process

TEA helps to understand the feasibility of new technologies/methods such as production of carbon-based products using eCO_2_R. Results from TEA assist in identifying pathways involving eCO_2_R which are economically competitive and can, therefore, be used on a large scale to produce carbon-based products. In all TEA studies, due to lack of data related to a real commercial eCO_2_R electrolyzer, a proton exchange membrane (PEM) or MEA H_2_O electrolyzer was used with identical general architecture and associated components. Additionally, an operational lifetime of 20 years was assumed for the CO_2_ conversion plant and capital equipment.

### 4A Feasibility Assessment for the Industrialization of eCO2R Products

Earlier study by Kabira et al. employs a CO_2_ cost of $40 t^–1^, an electrolyzer cost of $5000 m^–2^ and an electricity price of 2 cents kWh^–1^.^46^ The authors consider a 1.8 V cell voltage with 90% FE and an operating current density of 500 mA cm^–2^. Additionally, product separation costs are estimated at $10 t^–1^ for gaseous products and $60 t^–1^ for liquid products. With these parameters, the TEA study suggests that the levelized cost ($t^–1^) remains higher in comparison to the market prices for all examined eCO_2_R products (i.e., CO, HCOOH, C_2_H_5_OH and C_2_H_4_). The operational cost is found to be extremely sensitive to changes in electricity prices, with a 50% increase (4 cents) resulting in a 25% increase in the levelized cost for C_1_ products and more than a 33% increase for C_2_ products, respectively. The economic viability of these products is argued to depend on achieving current density, FE, and operational stability greater than 300 mA cm^2^, 80%, and 80,000 h, respectively, while keeping the cell voltage lower than 1.8 V.^46^

Verma et al., have introduced a gross-margin model to assess the technoeconomic feasibility of eCO_2_R products (specially C_1_ and C_2_ products) plugging maximum operating cell voltage (*V*_max_), minimum operating current density (*j*_min_), FE, and catalyst durability as key parameters.^141^ The results suggested that CO and HCOOH as eCO_2_R products are more economically viable products. A coproduction strategy via designing eCO_2_R system enable to produce C_2_ species with more economically viable products (CO, HCOOH) could be more beneficial in comparison to the single eCO_2_R product formation.

**Figure 3:**
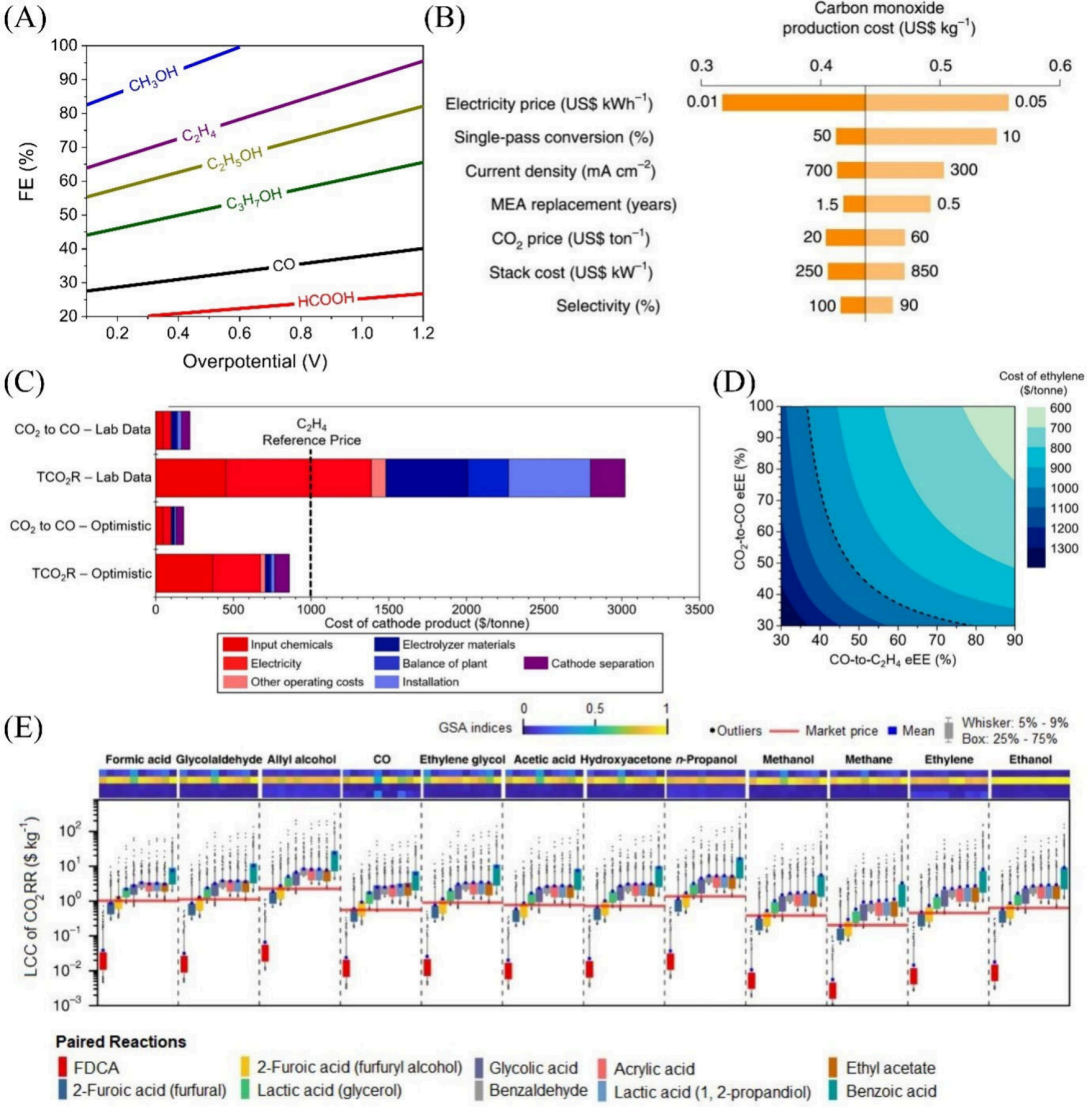
(A) The correlation between the end-of-life net present value (NPV) and overpotential and faradaic efficiency (FE) for C_3_H_7_OH, C_2_H_5_OH, CO, HCOOH, C_2_H_4_, and CH_3_OH products generated via eCO_2_R. The solid black line represents an NPV of $0 (IRR = 10%), with the region above the line yielding a positive NPV. The data extracted from the reference.^142^ (B) Analysis of the CO production cost depending on varying parameters including electricity price (US$ kWh^–1^), single-pass conversion (%), current density (mA cm^–2^), MEA replacement (years), CO_2_ price (US$ ton^–1^), stack cost ($ kW^–1^) and selectivity (%). Single-variable sensitivity analysis for the production cost of CO (US$0.44 kg^–1^).^37^ Reprinted with permission from ref (36). Copyright 2021, Springer Nature. (C) Comparison of C_2_H_4_ product cost via single step process and CO_2_–CO–C_2_H_4_ tandem CO_2_R process at laboratory level and optimistic level.^143^ Reprinted with permission from ref (142). Copyright 2021, American Chemical Society. (D) Cost of C_2_H_4_ production with varying electrical energy efficiency for CO_2_-to-CO and CO-to-C_2_H_4_ while all other inputs were kept fixed at their optimistic values. The dotted black lines in both (C) and (D) indicate C_2_H_4_ reference price ($1000 ton^–1^).^143^ Reprinted with permission from ref (142). Copyright 2021, American Chemical Society. (E) The levelized cost of chemicals (LCC) for eCO_2_R products based on different chemical products (2,5-furandicarboxylic acid (FDCA), 2-furoic acid (furfural, furfuryl alcohol), lactic acid (glycerol), glycolic acid, benzaldehyde, acrylic acid, lactic acid, ethyl acetate, and benzoic acid), produced via a coupled chemical oxidation reaction. The horizontal red line on each product indicates the market price.^144^ Reprinted with permission from ref (143). Copyright 2019, Springer Nature.

Oleksandr S. Bushuyev et al., conducted a TEA study with an electrolyzer cost of $500 kW^–1^ along with other predefined standard parameters. (e.g., energy conversion efficiency = 60%; F.E. = 90%; electricity cost = $0.02 kWh^–1^, and CO_2_ cost = $30 ton^–1^).^11^ The study suggests that higher carbon products [e.g., ethylene glycol (CH_2_OH)_2_ and C_3_H_7_OH] can be more attractive for commercialization due to their lower production value in comparison to the current market value.^11^ The study revealed that the electricity price and the CO_2_ cost are highly sensitive factors influencing the production cost variations of different eCO_2_R products. Jouny et al., performed TEA study to understand the feasibility of eCO_2_R process using a generalized CO_2_ electrolyzer system via calculating the end-of-life net present value (NPV) of various eCO_2_R products at 100 tons/day production rate.^142^ Under the given conditions, it has been concluded that production of CO and HCOOH offer a profitable route with NPVs of $13.5 million and $39.4 million, respectively. It has been argued that the cost of electricity is a decisive factor in the NPV of products. For instance, a minimal change (i.e., $0.01 kWh^–1^) resulted in a NPV difference of nearly $40 million for n-propanol production. Additionally, it has been highlighted that CO may achieve economic viability even at significantly low FE (<50%), provided the overpotential remains below 0.4 V (Figure 3A). Conversely, to render the production of CH_3_OH and C_2_H_5_OH profitable under a similar overpotential of 0.4 V, the FE must be higher than or equal to 60% and 90% or, respectively.

Gao et al., also performed TEA study for industrial CO_2_ electrolysis systems focusing on HCOOH, CO, C_2_H_5_OH and C_2_H_6_ products using different parameters.^139^ According to the analysis, the reported levelized cost for HCOOH ($0.468 kg^–1^) and CO ($0.449 kg^–1^) is relatively lower compared to production through conventional methods [HCOOH ($0.468 kg^–1^) and CO ($0.449 kg^–1^)]. In terms of capital expenditure (CAPEX), the cost of C_2_H_5_OH and C_2_H_6_ production has been observed to be higher than that of CO and HCOOH due to low FE (∼60% - C_2_H_5_OH and ∼60% - C_2_H_6_). To assess the sensitivity of FE, electricity price, cell voltage, single pass conversion efficiency (SPCE), and current density, these parameters were systematically varied by increasing and decreasing the amplitude by 25% from their base conditions. Notably, among all factors, cell voltage, electricity price, and FE were identified as significant influencers on the levelized cost. The 25% increase in selectivity reduces the levelized cost by ∼10.8% (HCOOH), ∼16% (CO), ∼18% (C_2_H_5_OH) and ∼19% (C_2_H_6_), respectively. Similarly, a 25% decrease in cell voltage results in ∼10.2% (HCOOH), ∼18% (CO), ∼34% (C_2_H_5_OH) and ∼13% (C_2_H_6_). A net present value (NPV) study reported that the production of C_1_ products (CO and HCOOH) can be profitable as early as third year (over a 20-year cycle) and succeeding in a NPV of $89.86 (HCOOH) and $52.11 million (CO) at the 20th year in the cycle. The C_2_ products under used conditions cannot be profitable at any stage throughout the 20-year life cycle and it requires. Similarly Shin et al.,^37^ studied the impact of different key metrices as discussed earlier on the economic feasibility of CO, HCOOH, C_2_H_5_OH, and C_2_H_6_ production. The study suggested that the price of electricity and cost of stack play an important role in reducing the cost of CO (Figure 3B) and other products. The CO and HCOOH products with production costs of $0.44 kg^–1^ and $0.59 kg^–1^, respectively, are economically feasible in comparison to the production of C_2_H_5_OH, and C_2_H_6_ formation. Based on these results, the ongoing focus should be directed toward the design of reactors, optimization of catalyst properties, and the reduction of energy loss to minimize cell voltage. This concerted effort is essential for improving selectivity and overall efficiency in eCO_2_R processes.

### 4B Comparative Competency Analysis of Different eCO_2_R Processes

The costs of the eCO_2_R products can vary significantly across different eCO_2_R processes. Spurgeon et al., compared the competence of four eCO_2_R processes: 1) Production of diesel fuel by electroreduction of CO_2_ to CO and subsequent use of the Fischer–Tropsch synthesis of liquid fuel (FTL) process to convert syngas to diesel fuel (CO_2_–CO-FTL); 2) One-step production of C_2_H_5_OH through the electroreduction of CO_2_ (CO_2_–C_2_H_5_OH); 3) Two-step production of C_2_H_5_OH by electroreduction of CO_2_ to CO and subsequent reduction of CO to C_2_H_5_OH (CO_2_–CO-C_2_H_5_OH); 4) Electroreduction of CO_2_ to HCOOH (CO_2_–HCOOH).^145^

The TEA study for the CO_2_–CO-FTL process, resulted in a base case LCF value of $18.9 gge^–1^ (gallon of gas equivalent) for diesel fuel (liquid end-product). With an assumed commercial diesel fuel price range of ∼ $2.12–3.19 gge^–1^, the base case LCF was found not to be economically competitive. However, the LCF value significantly reduced to $4.4 gge^–1^ for the case involving an optimistic performance parameter (optimistic case). For the case involving both the optimistic case and the monetary value for the carbon emissions offset ($100 per ton of CO_2_), the LCF reduced even further to $3.5 gge^–1^. Therefore, the case which resulted in the most economically competitive LCF value involved both aspirational but feasible performance parameters and a favorable policy environment. For the one step CO_2_–C_2_H_5_OH or two step CO_2_–CO-C_2_H_5_OH process, the base case LCF for C_2_H_5_OH was found to be $55.3 gge^–1^ and $49.8 gge^–1^ respectively. With the commercial ethanol fuel price range of ∼$2.10–2.55 gge^–1^, the base case requires considerable improvements to become economically competitive in both scenarios. The LCF values of ethanol for the optimistic case were obtained at $10.1 gge^–1^ and $8.2 gge^–1^ respectively, which are still high-pitched compared to commercially available ethanol. Finally, for the case involving both the optimistic case and the monetary value for carbon emissions offset, the LCF values reduced slightly to $8.6 gge^–1^ and $6.9 gge^–1^ for the one- and two-step process, respectively. The study suggests that both processes require substantial improvements in system and process parameters to become economically competitive. For the CO_2_–HCOOH process, the base case LCF for HCOOH was computed to be $1.16 kg^–1^ which was higher compared to commercially available HCOOH at the price range of ∼$0.4–0.6 kg^–1^. Here it should be noted that HCOOH is less suitable as a fuel due to its low energy density. For the optimistic case, the LCF reduced to a comparable value of $0.46 kg^–1^ making it economically competitive with commercial production processes. The LCF value was reduced even further to $0.36 kg^–1^ when the optimistic case was combined with a carbon emissions offset ($100 t^–1^ of CO_2_), making HCOOH production economically beneficial over commercially available HCOOH.

In another effort, four competing approaches to produce methyl formate (HCOOCH_3_) were analyzed.^146^ This study delves into the dynamics of CO_2_ capture processes in different electrolyzers, including a dual CH_3_OH/H_2_O electrolyzer equipped with a CO_2_ capture unit, a dual CH_3_OH/H_2_O electrolyzer directly fed with flue gas, a CH_3_OH/CH_3_OH electrolyzer with a CO_2_ capture unit, and a H_2_O/H_2_O electrolyzer producing HCOOH with a downstream HCOOH/CH_3_OH nonelectrochemical reactor. The results suggest that System 1 ($1.42 kg^–1^) and System 2 ($1.37 kg^–1^) exhibit a more economically favorable levelized cost compared to commercially produced HCOOCH_3_ ($1.60 kg^–1^). This advantage is primarily attributed to lower CH_3_OH and electricity consumption in Systems 1 and 2. In the scenario of the H_2_O/H_2_O electrolyzer with a downstream HCOOH/CH_3_OH nonelectrochemical reactor, the price of HCOOCH_3_ is relatively elevated at $2.70 kg^–1^. This is mainly due to additional costs associated with the HCOOH separation procedure and water utilized in the HCOOH/CH_3_OH nonelectrochemical reactor. The type of electrolyzers, such as PEM or alkaline electrolyzers, has demonstrated a minimal impact on the base levelized cost, and the trend remains consistent under both conditions. However, catholyte flow rate and current density exhibit modest impacts, while cathode methyl formate (HCOOCH_3_) FE, and the price of electricity play a significant role in determining the production cost of HCOOCH_3_. Debergh et al,^147^ performed TEA studies to compare the economic feasibility of syngas production via integrated capture with conversion and sequential methods. It has been noticed that syngas production via the integrated method is more economical than the sequential method due to lower direct air capture cost and higher CO_2_ utilization rate. Moreover, syngas production can be economically more feasible by operating a CO_2_ electrolyzer in a CO-selective mode and combining it with a separate PEM electrolyzer for H_2_ generation.^148^ Shun et al.^149^ discussed TEA of CO_2_ industrial electrolysis using both an electro-stack and a single-chamber electrolyzer (SC electrolyzer) for CO_2_-to-CO electrolysis. The findings reveal that CO can be produced at a lower cost using the electro-stack electrolyzer ($0.37 kg^–1^) compared to the SC electrolyzer ($0.41 kg^–1^). For a CO production capacity of 50,000 kg/day, the plant utilizing the SC electrolyzer would incur a loss of approximately $3,000,000, whereas the plant employing an electrostack (three electrolyzer stacks) would generate a profit of about $5,600,000 over 10 years of operation. The study suggests that the electro-stack configuration could be more attractive for the industrialization of the eCO_2_R process. Sisler et al.^143^ compared the ethylene production in a neutral MEA, an alkaline flow cell electrolyzer via single step process and CO_2_–CO–C_2_H_4_ tandem process using a high-temperature solid-oxide electrolyzer cell (SOEC) for CO production and neutral MEA or an alkaline cell for CO to C_2_H_4_ conversion. The results suggest that under optimistic conditions (e.g., CO_2_-to-CO, cell voltage; −1.3 V, FE; 100%, current density; 1000 mA cm^–2^ and CO-to-C_2_H_4_, cell voltage; −1.8 V, FE; 90%, current density; 1000 mA cm^–2^), the C_2_H_4_ can be produced below the reference price ($1,000/tonne) via the CO_2_–CO–C_2_H_4_ tandem process (Figure 3C). The overall economic feasibility depends on the electrical energy efficiencies (eEEs) of both steps (Figure 3D). The study highlights that CO_2_ → CO eEE of 80% is achievable but CO → C_2_H_4_ eEE should be increased to at least 40% to make process practically economically feasible.

### 4C Role of Coupled Valuable Oxidation Products

The economic viability of eCO_2_R can be enhanced by coupling it with other anodic processes that have lower thermodynamic cell potentials in comparison to the oxygen evolution reaction (OER) and produce additional valuable products.^150,151^ In recent study, Wang et al. performed eCO_2_R coupled with allyl alcohol oxidation reaction to acrolein on anode and observed 0.7 V decrease in the full-cell voltage of the system resulting 1.6× less energy consumption in comparison to the most efficient CO_2_-to-CO eCO_2_R process.^152^ Similarly, CO-to-C_2_H_4_ required 55% less energy when OER was replaced by glycerol oxidation reaction.^153^ by H_2_-integrated CO_2_ reduction reaction result in ∼42% reduction in energy consumption and potentially be economically more feasible process for industrialization.^154^ A study by Verma et al. demonstrated that pairing eCO_2_R with glycerol electro-oxidation can reduce energy consumption by up to 53% compared to using OER.^155^ This approach lowers the onset cell potentials for producing HCOOH, C_2_H_4_, and C_2_H_5_OH significantly to −0.9 V, −0.95 V, and −1.3 V, respectively, compared to −1.75 V, −1.8 V, and −2.1 V when using OER. In another work, ∼35% reduction in energy consumption has been observed when, the electroreduction of CO_2_ to CO was paired with the electrooxidation of 1,2-propanediol to lactic acid.^156^ Na et al. research highlighted that coupling eCO_2_R with organic oxidation reactions (OORs), especially with 2,5-furandicarboxylic acid (FDCA) and 2-furoic acid, makes all eCO_2_R products economically feasible (Figure 3E).^144^ Although the levelized cost of chemicals (LCC)-to-market price ratio is low due to current market prices ranging from $32 to $580 per kg, the economic feasibility remains intact as long as the market price of FDCA remains above $4.25 kg^–1^.

### 4D Other Studies

Most TEA reports have generally overlooked the interdependence among the current density, FE, and cell voltage in their analyses. To address this gap, Bagemihl et al., conducted a multiscale modeling approach, encompassing the process, the electrolyzer, and the channel scale for the eCO_2_R process. Their study explored the impact of this comprehensive modeling on the economic feasibility of eCO_2_R products.^35^ A case study focusing on ethylene as the primary product with a target production rate of 10,000 kg/day has been discussed. The study contributes significantly by modeling the relationships among various metrics (e.g., current density, CO_2_ consumption, flow rate, and others), highlighting the interdependency of these metrics. This approach plays a crucial role in identifying ideal conditions for the commercialization of eCO_2_R by analyzing the trade-off between the investment and operating costs. However, it is essential to note that the study makes several assumptions in the process modeling, such as discounting gaseous mass transport in the porous gas diffusion layer, assuming CO_2_ equilibrium at the gas–liquid interface, migration of bicarbonate/carbonate ions, and lower electricity price ($0.03 kWh^–1^), which might deviate the study from a more realistic scenario. Here it should be noted that the electricity price can be substantially lowered due to progress in the field of renewable energy resources; however, it may swing depending on the economy and policy related to the manufacturing and installation of renewable energy resources.^157^ Integrating photovoltaic (PV) and CO_2_ electrolyzers can have benefit for on-demand chemicals manufacture and usage of solar electricity directly for CO_2_ conversion. Thus, a process level TEA study has been performed for PV operated CO_2_-to-CO conversion with varying different parameters (e.g., the membrane cost, solar to chemical efficiency, F.E., and current density). The analysis suggests that with a >80 mA cm^–2^ current density or 22% CO_2_ conversion, CO_2_-to-CO process can be an economically viable process over the conventional CO production process in a 4 MW production plant.^158^ However, the study is limited to CO formation, and the analysis for other higher carbon products has not been discussed. Crandall et al. conducted a TEA to assess the feasibility of employing electrochemical acetate instead of glucose in the food and chemical industries. Remarkably, their observations indicate that electrochemical acetate could positively impact the production cost, resulting in a 16% decrease for both food and chemical production. The availability of inexpensive electricity is anticipated to further contribute to substantial reductions in production costs.^159^

**Figure 4:**
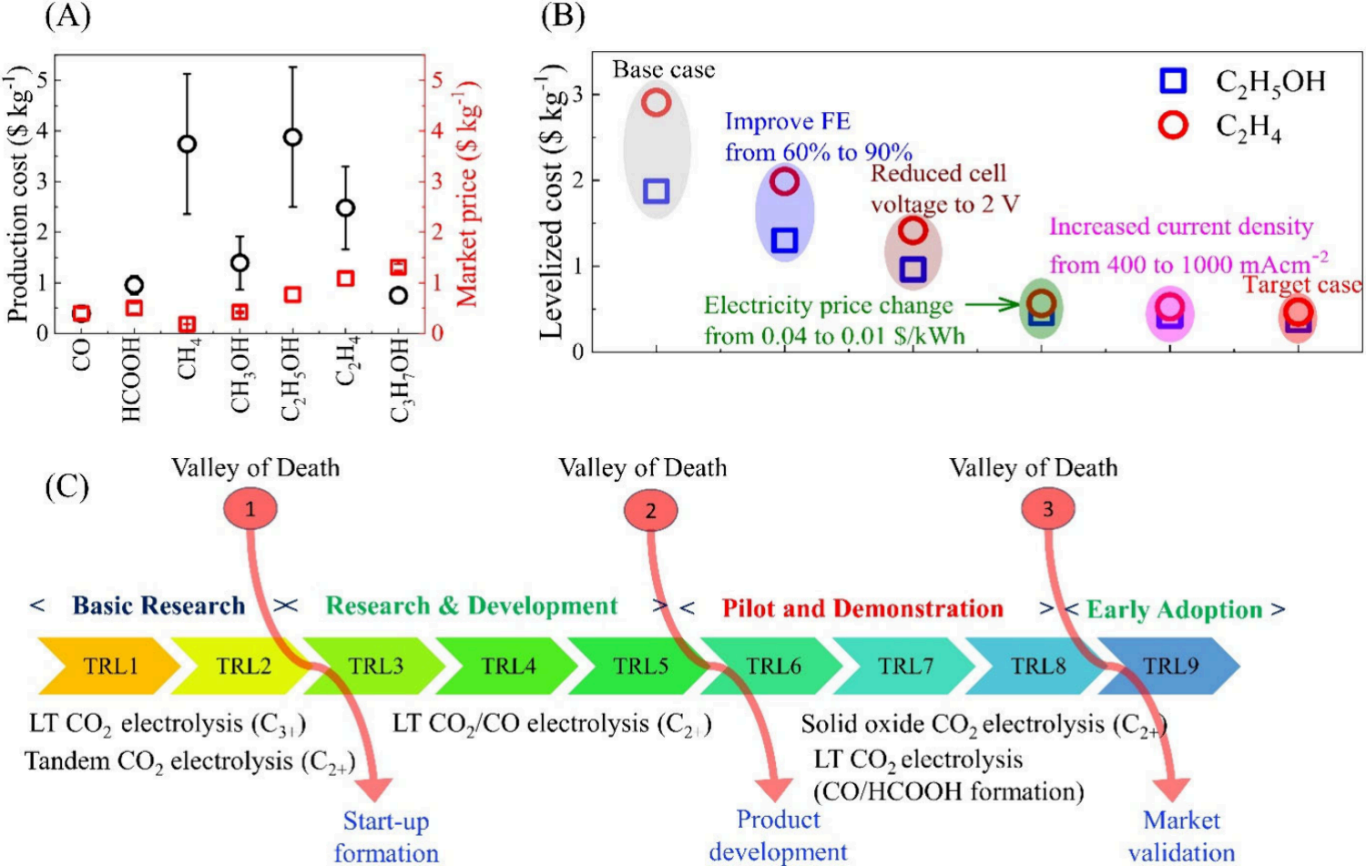
(A) Average market prices and average production costs are based on different TEA studies. The data has been extracted from references^10,11,46,140,142,145,146,160−162^ (B) Strategies to accomplish the competitive prices for C_2_H_5_OH and C_2_H_4_. The data has been taken from^139^ and (C) Technology readiness levels (TRL) of different CO_2_ electrolysis processes.^127,163^ Solid oxide CO_2_ electrolysis and low temperature CO_2_ electrolysis to C_1_ are the leading technologies to be commercialized earlier.

The comparison of market prices and the average base-case levelized cost values collected from different eCO_2_R TEA studies suggests that CO and HCOOH production is more commercially feasible at present than other eCO_2_R products (Figure 4A).^10,11,46,140,142,145,146,160−162^ This outcome is partially due to the high current density (>300 mA cm^–2^) with a simultaneously high FE (>90%) that has been achieved in CO and HCOOH production, as well as low number of electrons (2e^–^) required per product molecule. Some TEA have suggested that other eCO_2_R products can also compete with commercially available products if certain eCO_2_R metrics are met (e.g., current density >300 mA cm^–2^, cell voltage <2.5 V, FE > 80%, lifetime >50,000 h, etc.).^15^ For example, in one study, improving the FE of C_2_H_5_OH and C_2_H_4_ from 60% to 90% resulted in a ∼30% decrease in the levelized cost of C_2_H_5_OH and C_2_H_4_. A further decrease in the cell voltage and an improvement in the current density resulted in a levelized cost for C_2_H_5_OH and C_2_H_4_ production via the eCO_2_R process comparable to the market price (Figure 4B).^139^

In considering the many TEA that have been reported for eCO_2_R systems, it is illustrative to consider the current state-of-the-art for different types of CO_2_ electrolysis in regard to their technology readiness levels (TRL) (Figure 4C). Electrolyzers for CO and HCOOH production via the eCO_2_R have progressed beyond the early conceptual technology “valleys of death” and are facing challenges related to scaling up, controlling units, manufacturing catalysts at larger scales, and other challenges for early adoption such as market acceptance (Figure 4C). However, other emerging technologies such as low-temperature (LT) CO_2_ electrolysis to C_2+_ products and tandem electrolysis processes are in their early stages and consist of more complex reactions and lacking a reliable catalytic system to durably achieve the targeted current density (>300 mA cm^–2^), high FE (>80%), and long operational lifetime (>50,000h). Significant future effort should thus be directed toward developing high-performance eCO_2_R to C_2+_ products at the required metrics, as determined by TEA for market competitiveness. An in-depth understanding of the C_2+_ reaction mechanisms via in situ spectroscopy/microscopy and inoperando techniques combined with computational study and machine learning models may provide crucial insight into design new high-selectivity catalysts. Furthermore, knowledge related to electrolyzer design and operating challenges gained from industrial CO and HCOOH production may be translatable to aid in overcoming technological commercialization challenges for multicarbon products.

## Supporting Information Available

Performance metrics of various electrochemical CO_2_ reduction reaction processes (Table S1) (PDF)
Literature review on TEA studies for electrochemical CO_2_ reduction reaction processes (Table S2) (PDF)
