# Supplementary material for: Electrochemical CO2 Conversion Commercialization Pathways: A Concise Review on Experimental Frontiers and Technoeconomic Analysis
Source: Environ Sci Technol Lett. 2024 Sep 17;11(11):1161–74. doi: 10.1021/acs.estlett.4c00564 (PMC11562736; doi:10.1021/acs.estlett.4c00564)
Supplement: Supplementary file 2 — ez4c00564_si_002.pdf [file ez4c00564_si_002.pdf]

Supporting Information for

Electrochemical CO_2 Conversion Commercialization Pathways: A Concise Review on Experimental Frontiers and Techno-Economic Analysis

Bijandra Kumar[a], Baleeswaraiah Muchharla[a], Moumita Dikshit[b], Saudagar Dongare[c], Kapil Kumar[b], Burcu Gurkan[c], and Joshua M. Spurgeon[d]*

[a] *Department of Math. Comp. Science and Eng. Technology, Elizabeth City State University, Elizabeth City, NC 27909 USA*

[b]*Laboratory of Environmental Sustainability and Energy Research (LESER), National Institute of Technology Delhi, New Delhi, 110036 India*

[c]*Department of Chemical and Biomolecular Engineering, Case Western Reserve University, Cleveland, OH 44106 USA*

[d]*Conn Center for Renewable Energy Research, University of Louisville, Louisville, KY, 40292 USA*

*Email: bkumar@ecsu.edu

Table S1. Summary of various electrochemical CO2 reduction eCO_2R) reaction processes products consisting of high FE and partial current density. We initiated our investigation by examining reports that highlighted very high current densities, specifically those exceeding 1000 mA/cm² and high FE. After an initial review, we broadened our scope to include studies with current densities ranging from 100 to 1000 mA/cm². This strategy ensured a thorough compilation of studies with diverse current densities, which is crucial for understanding variations in electrochemical reduction processes.

**Product**	**Catalyst**	**Electrolyte**	**Cell type**	**FE (%)**	**Cell voltage (V)**	**Partial current density (mA cm^{-2})**	**Stability (hr)**	**Reference**
CO	Ag/carbon nanotubes	1 M KOH	Flow cell	95	3	350		1
	Ag/carbon paper	3 M KOH	Flow cell	100	2.75	343		2
	Ag GDE	1.5 M $KHCO_3$	Flow cell	70	5	210		3
	O2 plasma treated Ag	0.01 M CsOH	MEA	80	4	500	100	4
	Coral Ag	0.5 M $KHCO_3$	MEA	90	2.75	312	30	5
	3D AuAg	1 M $KHCO_3$	MEA	76	4.1	302	70	6
	Ni-SA-NCs	0.5 M $KHCO_3$	MEA	98	2.6	145	9	7
	Ni-N/C	0.1 M $KHCO_3$	MEA	93	3	113.6		8
	PSMIM-PBI	0.01 mM $KHCO_3$	Flow cell	98	3	140	4380	9
	Au/C	0.1 M $KHCO_3$	Flow cell	85	3	425	100	10
	Hg-CoTPP/NG	1 M KOH	Flow cell	98.7	0.65 V vs RHE	420	360	11

HCOOH	SnO_2/C-NPs	0.1 M $KHCO_3$, adjusted with KOH to a pH value of 10	Flow cell	83	3.08	163	3	12
	Sn/Carbon paper	0.5 M Na_2CO_3 + 0.5 M Na_2SO_4	H-cell	80	1.6 V vs Ag/AgCl	300		13
	PtRu alloy/carbon paper	0.5 M K_2SO_4	Flow cell	96	0.82 V vs Ag/AgCl	143		14
	carbon-supported Pb and PtRu	0.5 M K_2SO_4	Flow cell	85	2 V vs Ag/AgCl	45	8.3	15
	Sn NPs/GDE	acidic ion-exchange bead electrolyte media	Flow cell	94	3.5	140	500	16
	Bi MOF	2.0 M $KHCO_3$		93	0.8 V vs RHE	55	43	17
	Bi@NCFs	0.5 M $KHCO_3$	Flow cell	81.3	1.3 V vs RHE	37.5	48	18
	GDE-supported j-BiNSs	0.5 M $KHCO_3$	Flow cell	90	1 V vs RHE	117	72	19
	In NSs	1 M KOH	Flow cell	96.3	1 V vs RHE	360	140	20

	Sn(S)-H	0.5 M $K_2SO_4 + H_2SO_4$ (pH = 3)	three electrode flow cell	73	2.5 V vs RHE	730.2	13.5	21
	Sn(S)-H	0.5 M $K_2SO_4 + H_2SO_4$ (pH = 3)	three electrode flow cell	89.8	2.5 V vs RHE	359.2	13.5	21
	$ZnIn_2S_4$	1 M $KHCO_3$	Flow cell	99.3	1.2 V vs RHE	300	60	22
	nBuLi-B	1 M $KHCO_3$	three electrode flow cell	92	1.05 V vs RHE	460	100	[23]
	SnCu/SnOx core-shell NPs	1 M KOH	H-cell	88	0.93 V vs RHE	357.9	40	[24]
	SnCu/SnOx core-shell NPs	1 M KOH	H-cell	90	0.93 V vs RHE	218.8	40	[24]
	Bismuth oxide nanotubes	1 M KOH	H-cell	97	1.05 V vs RHE	279.3	48	[25]
	SnO_2/C	0.1 M H_2SO_4 + 0.4 M K_2SO_4	Flow cell	88	1.52 V vs RHE	314	4	[26]
CH_3OH	$Rh1Cu_4$	1 M KOH	Flow cell (CO as feed gas)	46.2	0.95 V vs RHE	111.7	120	[27]
	Cu_2NCN	0.5 M $KHCO_3$	MEA	48	2.8	92.3	10.5	[28]

	imine-bridged covalent organic nanosheets (imine-CONs) from the polymerization of CoTAPc	0.2 M KOH and 1.5 M KCl	Flow cell	38.7	0.78 V vs RHE	91.7	6	[29]
	Ag,S-Cu_2O/Cu	$BMImBF_4/H_2O$ (1:3)	H-type cell	67.4	1.18 V vs RHE	82.7	24	[30]
	Sn1/Vo-CuO-90	$BMImBF_4/H_2O$ (1:3)	H-type cell	88.6	2.0 V vs. Ag/Ag+	59.4	36	[31]
	CuSAs/TCNFs	0.1 M $KHCO_3$	H-type cell	44	0.9 V vs RHE	93	50	[32]
	Pd−Cu bimetallic aerogel	$BMImBF_4/H_2O$ (1:3)		80	2.1 V vs. Ag/Ag+	31.8		[33]
CH_4	Porus Cu	0.3 M $KHCO_3$	Flow cell	70	5.4	500	12	[34]
	Cu(I)-based co-ordination polymer	1 M KOH	three-channel flow cell	79	0.9 V vs RHE	370	5.0	[35]

	Low coordination Cu + CNP additives	An anion exchange membrane (Sustainion X37–50)was used as the solid cathode electrolyte.	MEA	62	4	136	110	[36]
	EDTMPA adsorbed on Cu(110)	1 M KOH	Flow cell	64	1 V vs RHE	192	6	[37]
	Cu-np/NC	1 M KOH	Flow cell	65.8	4	234.9	50	[38]
	Sputtered Cu	1.5 M $KHCO_3$	H-cell	48	1 V vs RHE	120	14	[39]
	Sputtered Cu	1 M $KHCO_3$	Flow cell	48	1.4 V vs RHE	108	22	[40]
	$Ag@Cu_2O$	1 M KOH	Flow cell	74	1.2 V vs RHE	178	5.5	[41]
	Porus Cu	3 M $KHCO_3$	MEA	27	2 V vs Ag/AgCl	128		[42]
CH_3COO	Cu NSs	2 M KOH	Flow cell	48	0.75	131	3	[43]
	$Cu(OH)_2$-derived Cu/CuOx	0.5 M KCl + 1 M KOH	H-cell	71	1.7 V vs Ag/AgCl	86	20	[44]
	Mo_8O_x/Cu	saturated $NaHCO_3$	Flow cell	48.68	1.13	53.5	3.1	[45]

	PcCu-TFPN	0.1 M $KHCO_3$	Flow cell	90.3	0.8	11.3	85	[46]
	Cu–CuI	1 M KOH	Flow cell	5	1	44.7	85	[47]
	CuPd	1 M KOH	Flow cell	70	1.03	425	100	[48]
	Cu(I)-benzimidazole	3 M KOH	Flow cell	61	0.59	244	250	[49]
	Cu49Pd51	1 M KOH	Flow cell	65	1	130	30	[50]
	Cu NC	PSE	custom cell	43	2.2	193.5	150	[51]
	N-doped nanodiamond/Si rod array	0.5 M $NaHCO_3$		75	0.8		3.1	[52]
	CuAu	1 M KOH	Flow cell (CO as feed gas)	39		217	2.5	[53]
	N-Cu	2 M KOH	Flow cell (CO as feed gas)	42	1.27	180		[54]
C_2H_5OH	MoP-Im	3 M KOH and 3 M KCl	Flow cell	77.4	0.5	54.9	112	[55]
	carbon-supported copper	0.1 M $KHCO_3$		91	0.7	1.2	16	[56]
	Ag-Co_3O_4-CeO_2/LGC	0.1 M $KHCO_3$	H-cell	54.2	0.36	6.9376	60	[57]

	SnS_2 nano sheets	0.5 M $KHCO_3$	Flow cell	82.5	0.9	14.685	100	[58]
	N-C/Cu	1 M KOH	MEA	52	0.68	156	15	[59]
	dCu_2O/Ag 2.3%	4 M KCl	Flow cell	40.8	0.87	326.4		[60]
	CuDAT-wire	1 M KOH	Flow cell	27.3	0.69	275	8	[61]
	CuAg alloy film	1 M KOH	Flow cell	25.9	0.7	310.8		
	Cu_2O HNCs	3 M KOH		8.2	1.6	44.28		[62]
	Cu9Ga4	1 M KOH	Flow cell	28	1.8	337.96		[63]
	F−Cu	1 M KOH	Flow cell	16	0.75	256	40	[64]
C_2H_4	Cu nanoparticles	1 M KOH	Flow cell	46	0.7	150	4	[65]
	Cu/carbon paper	0.1 M KBr	Flow cell	57		170	2.5	[66]
	Cu/Graphite, carbon	7 M KOH	Flow cell	70	0.55	275	150	[67]
	Cu/carbon paper	1 M KOH	Flow cell	45	0.66	474	2	[68]
	CuAg alloy/carbon paper	1 M KOH	Flow cell	58	0.6	175		[69]
	Cu	0.15 M $KHCO_3$	Flow cell	58	3.7	350	65	[70]
	Cu-CIPH	7 M KOH	Flow cell	60	3.23	930	60 in 0.1 M KHCO3	[71]

	CuONPs-1.7/GDE	1 M KOH	Flow cell	50	1.8 V vs Ag/AgCl	1000	1.5	[72]
	CuO/ZnO/C	1 M KOH	Flow cell	50.9	0.75	186.8	75	[73]
	F−Cu	1 M KOH	Flow cell	63	0.75	1008	40	[64]
	CuNCs	1 M KOH containing 0.2 M CsI	Flow cell	46.6	0.65	245.1	16	[74]
	Cu nanocubes	0.1 M $KHCO_3$	MEA	43	3.5	90	6	[75]
	CuO nanoplate arrays	0.5 M KCl	Flow cell	84.5	0.81	84.5	55	[76]
	Cu9Ga4	1 M KOH	Flow cell	30	1.8	362.1		[63]
C_3H_7OH	Pb-doped Cu	1 M KOH	Flow cell (CO as feed gas)	47	0.68	60	110	[77]
	CuAg alloy			56.7		59.3		[78]
	CuS NSs	0.1 M $KHCO_3$	H-cell	15.4	1.05	9.9	10	[79]
	Cu_2O HNCs	3 M KOH		8.2	1.1	44.28		[62]
	Ag-Ru-Cu	1 M KOH	MEA (CO as feed gas)	37		111	102	[80]
	CuO/SiO_2	1 M KCl	Flow cell	13.3	2.38	94		[81]

C_3H_6	CuNCs	1 M KOH containing 0.2 M CsI	Flow cell	1.42	0.6 V vs RHE	4	16	[74]
	Cu nanocubes	0.1 M $KHCO_3$	MEA	1	3.5	3	6	[75]

Table S2. Summary of literature on Techno-Economic Analysis (TEA) studies for eCO_2R reaction processes.

S. No.	Title	Author	Year	Ref
1	A Gross-Margin Model for Defining Technoeconomic Benchmarks in the Electroreduction of CO2.	Verma, S., et al.,	2016	[82]
2	Assessment of Solar-to-Fuels Strategies: Photocatalysis and Electrocatalytic Reduction.	Herron, J.A. and C.T. Maravelias,	2016	[83]
3	Terawatt-scale photovoltaics: Trajectories and challenges.	Haegel, N.M., et al.,	2017	[84]
4	What Should We Make with CO2 and How Can We Make It?	Bushuyev, O.S., et al.,	2018	[85]
5	General Techno-Economic Analysis of CO2 Electrolysis Systems. Industrial & Engineering Chemistry Research.	Jouny, M., W. Luc, and F. Jiao,	2018	[86]
6	A comparative technoeconomic analysis of pathways for commercial electrochemical CO2 reduction to liquid products.	Spurgeon, J.M. and B. Kumar,	2018	[87]
7	Technologies and infrastructures underpinning future CO2 value chains: A comprehensive review and comparative analysis.	Jarvis, S.M. and S. Samsatli,	2018	[88]
8	What would it take for renewably powered electrosynthesis to displace petrochemical processes?	De Luna, P., et al.,	2019	[89]
9	Electrochemical CO_2 Reduction into Chemical Feedstocks: From Mechanistic Electrocatalysis Models to System Design.	Kibria, M.G., et al.,	2019	[90]
10	Co-electrolysis of CO2 and glycerol as a pathway to carbon chemicals with improved technoeconomics due to low electricity consumption.	Verma, S., S. Lu, and P.J.A. Kenis,	2019	[91]
11	Electroreduction of CO2 to CO Paired with 1,2-Propanediol Oxidation to Lactic Acid. Toward an Economically Feasible System.	Pérez-Gallent, E., et al.,	2019	[92]

12	General technoeconomic analysis for electrochemical coproduction coupling carbon dioxide reduction with organic oxidation.	Na, J., et al.,	2019	93
13	High-Pressure Electrochemical Reduction of CO2 to Formic Acid/Formate: Effect of pH on the Downstream Separation Process and Economics.	Ramdin, M., et al.,	2019	94
14	A techno-economic evaluation approach to the electrochemical reduction of CO2 for formic acid manufacture.	Rumayor, M., et al.,	2019	95
15	A perspective on practical solar to carbon monoxide production devices with economic evaluation.	Chae, S.Y., et al.,	2020	96
16	Techno-economic assessment of low-temperature carbon dioxide electrolysis.	Shin, H., K.U. Hansen, and F. Jiao,	2021	97
17	Tandem and Hybrid Processes for Carbon Dioxide Utilization.	Overa, S., et al.,	2021	98
18	Coupling electrochemical carbon dioxide conversion with value-added anode processes: An emerging paradigm.	Vass, Á., B. Endrődi, and C. Janáky,	2021	99
19	Glycerol Oxidation Pairs with Carbon Monoxide Reduction for Low-Voltage Generation of C2 and C3 Product Streams.	Yadegari, H., et al.,	2021	100
20	Comparative Technoeconomic Analysis of Pathways for Electrochemical Reduction of CO2 with Methanol to Produce Methyl Formate.	Spurgeon, J.M., et al.,	2022	101
21	Accelerating CO(2) electrochemical conversion towards industrial implementation.	Segets, D., C. Andronescu, and U.P. Apfel,	2023	102
22	Techno-economic Assessment of CO2 Electrolysis: How Interdependencies between Model Variables Propagate Across Different Modeling Scales.	Bagemihl, I., et al.,	2023	103
23	Techno-economic Analysis and Carbon Footprint Accounting for Industrial CO2 Electrolysis Systems.	Gao, T., et al.,	2023	104
24	Turning Carbon Dioxide into Sustainable Food and Chemicals: How Electrosynthesized Acetate Is Paving the Way for Fermentation Innovation.	Crandall, B.S., et al.,	2023	105
25	Coupling electrochemical CO2 reduction with value-added anodic oxidation reactions: progress and challenges.	Li, Y. and T.-B. Lu,	2024	106
26	Efficient CO and acrolein co-production via paired electrolysis.	Wang, X., et al.,	2024	107

27	Integrating hydrogen utilization in CO2 electrolysis with reduced energy loss.	Jiang, X., et al.,	2024	108

1. S. Ma, R. Luo, J. I. Gold, A. Z. Yu, B. Kim and P. J. A. Kenis, *Journal of Materials Chemistry A*, 2016, **4**, 8573-8578.
2. S. Verma, X. Lu, S. Ma, R. I. Masel and P. J. A. Kenis, *Physical Chemistry Chemical Physics*, 2016, **18**, 7075-7084.
3. T. Haas, R. Krause, R. Weber, M. Demler and G. Schmid, *Nature Catalysis*, 2018, **1**, 32-39.
4. K. Ye, G. Zhang, X.-Y. Ma, C. Deng, X. Huang, C. Yuan, G. Meng, W.-B. Cai and K. Jiang, *Energy & Environmental Science*, 2022, **15**, 749-759.
5. W. H. Lee, Y.-J. Ko, Y. Choi, S. Y. Lee, C. H. Choi, Y. J. Hwang, B. K. Min, P. Strasser and H.-S. Oh, *Nano Energy*, 2020, **76**, 105030.
6. A. Ozden, Y. Liu, C.-T. Dinh, J. Li, P. Ou, F. P. García de Arquer, E. H. Sargent and D. Sinton, *ACS Applied Energy Materials*, 2021, **4**, 7504-7512.
7. H.-Y. Jeong, M. Balamurugan, V. S. K. Choutipalli, E.-s. Jeong, V. Subramanian, U. Sim and K. T. Nam, *Journal of Materials Chemistry A*, 2019, **7**, 10651-10661.
8. D. Kim, W. Choi, H. W. Lee, S. Y. Lee, Y. Choi, D. K. Lee, W. Kim, J. Na, U. Lee, Y. J. Hwang and D. H. Won, *ACS Energy Letters*, 2021, **6**, 3488-3495.
9. R. B. Kutz, Q. Chen, H. Yang, S. D. Sajjad, Z. Liu and I. R. Masel, *Energy Technology*, 2017, **5**, 929-936.
10. Z. Yin, H. Peng, X. Wei, H. Zhou, J. Gong, M. Huai, L. Xiao, G. Wang, J. Lu and L. Zhuang, *Energy & Environmental Science*, 2019, **12**, 2455-2462.
11. M. Fang, L. Xu, H. Zhang, Y. Zhu and W.-Y. Wong, *Journal of the American Chemical Society*, 2022, **144**, 15143-15154.
12. D. Kopljar, N. Wagner and E. Klemm, *Chemical Engineering & Technology*, 2016, **39**, 2042-2050.
13. S. Sen, B. Skinn, T. Hall, M. Inman, E. J. Taylor and F. R. Brushett, *MRS Advances*, 2017, **2**, 451-458.
14. X. Lu, D. Y. C. Leung, H. Wang, M. M. Maroto-Valer and J. Xuan, *Renewable Energy*, 2016, **95**, 277-285.
15. X. Lu, D. Y. C. Leung, H. Wang and J. Xuan, *Applied Energy*, 2017, **194**, 549-559.
16. H. Yang, J. J. Kaczur, S. D. Sajjad and R. I. Masel, *Journal of CO2 Utilization*, 2017, **20**, 208-217.
17. N. Li, P. Yan, Y. Tang, J. Wang, X.-Y. Yu and H. B. Wu, *Applied Catalysis B: Environmental*, 2021, **297**, 120481.
18. H. Li, K. Ao, J. Liu, F. Sun, X. Yu, X. Zhang, J. Shi, X. Yue and J. Xiang, *Chemical Engineering Journal*, 2023, **464**, 142672.
19. L.-W. Chen, Y.-C. Hao, J. Li, L. Hu, X. Zuo, C. Dai, Z.-L. Yu, H.-Z. Huang, W. Tian, D. Liu, X. Chang, P. Li, R. Shao, B. Wang and A.-X. Yin, *Small*, 2023, **19**, 2301639.
20. S.-H. Li, S. Hu, H. Liu, J. Liu, X. Kang, S. Ge, Z. Zhang, Q. Yu and B. Liu, *ACS Nano*, 2023, **17**, 9338-9346.
21. H. Shen, H. Jin, H. Li, H. Wang, J. Duan, Y. Jiao and S.-Z. Qiao, *Nature Communications*, 2023, **14**, 2843.

22. L.-P. Chi, Z.-Z. Niu, X.-L. Zhang, P.-P. Yang, J. Liao, F.-Y. Gao, Z.-Z. Wu, K.-B. Tang and M.-R. Gao, *Nature Communications*, 2021, **12**, 5835.
23. L. Fan, C. Xia, P. Zhu, Y. Lu and H. Wang, *Nature Communications*, 2020, **11**, 3633.
24. K. Ye, Z. Zhou, J. Shao, L. Lin, D. Gao, N. Ta, R. Si, G. Wang and X. Bao, 2020, **59**, 4814-4821.
25. Q. Gong, P. Ding, M. Xu, X. Zhu, M. Wang, J. Deng, Q. Ma, N. Han, Y. Zhu, J. Lu, Z. Feng, Y. Li, W. Zhou and Y. Li, *Nature Communications*, 2019, **10**, 2807.
26. J. Gu, S. Liu, W. Ni, W. Ren, S. Haussener and X. Hu, *Nature Catalysis*, 2022, **5**, 268-276.
27. J. Zhang, P. Yu, C. Peng, X. Lv, Z. Liu, T. Cheng and G. Zheng, *ACS Catalysis*, 2023, **13**, 7170-7177.
28. S. Kong, X. Lv, X. Wang, Z. Liu, Z. Li, B. Jia, D. Sun, C. Yang, L. Liu, A. Guan, J. Wang, G. Zheng and F. Huang, *Nature Catalysis*, 2023, **6**, 6-15.
29. Y. Song, P. Guo, T. Ma, J. Su, L. Huang, W. Guo, Y. Liu, G. Li, Y. Xin, Q. Zhang, S. Zhang, H. Shen, X. Feng, D. Yang, J. Tian, S. K. Ravi, B. Z. Tang and R. Ye, *Advanced Materials*, **n/a**, 2310037.
30. P. Li, J. Bi, J. Liu, Q. Zhu, C. Chen, X. Sun, J. Zhang and B. Han, *Nature Communications*, 2022, **13**, 1965.
31. W. Guo, S. Liu, X. Tan, R. Wu, X. Yan, C. Chen, Q. Zhu, L. Zheng, J. Ma, J. Zhang, Y. Huang, X. Sun and B. Han, *Angewandte Chemie International Edition*, 2021, **60**, 21979-21987.
32. H. Yang, Y. Wu, G. Li, Q. Lin, Q. Hu, Q. Zhang, J. Liu and C. He, *Journal of the American Chemical Society*, 2019, **141**, 12717-12723.
33. L. Lu, X. Sun, J. Ma, D. Yang, H. Wu, B. Zhang, J. Zhang and B. Han, *Angewandte Chemie International Edition*, 2018, **57**, 14149-14153.
34. C. A. Obasanjo, G. Gao, J. Crane, V. Golovanova, F. P. García de Arquer and C.-T. Dinh, *Nature Communications*, 2023, **14**, 3176.
35. L. Zhang, X.-X. Li, Z.-L. Lang, Y. Liu, J. Liu, L. Yuan, W.-Y. Lu, Y.-S. Xia, L.-Z. Dong, D.-Q. Yuan and Y.-Q. Lan, *Journal of the American Chemical Society*, 2021, **143**, 3808-3816.
36. Y. Xu, F. Li, A. Xu, J. P. Edwards, S.-F. Hung, C. M. Gabardo, C. P. O'Brien, S. Liu, X. Wang, Y. Li, J. Wicks, R. K. Miao, Y. Liu, J. Li, J. E. Huang, J. Abed, Y. Wang, E. H. Sargent and D. Sinton, *Nature Communications*, 2021, **12**, 2932.
37. Z. Han, D. Han, Z. Chen, J. Gao, G. Jiang, X. Wang, S. Lyu, Y. Guo, C. Geng, L. Yin, Z. Weng and Q.-H. Yang, *Nature Communications*, 2022, **13**, 3158.
38. Y. Wu, C. Chen, X. Yan, R. Wu, S. Liu, J. Ma, J. Zhang, Z. Liu, X. Xing, Z. Wu and B. Han, *Chemical Science*, 2022, **13**, 8388-8394.
39. A. Sedighian Rasouli, X. Wang, J. Wicks, G. Lee, T. Peng, F. Li, C. McCallum, C.-T. Dinh, A. H. Ip, D. Sinton and E. H. Sargent, *ACS Sustainable Chemistry & Engineering*, 2020, **8**, 14668-14673.
40. X. Wang, A. Xu, F. Li, S.-F. Hung, D.-H. Nam, C. M. Gabardo, Z. Wang, Y. Xu, A. Ozden, A. S. Rasouli, A. H. Ip, D. Sinton and E. H. Sargent, *Journal of the American Chemical Society*, 2020, **142**, 3525-3531.

41. L. Xiong, X. Zhang, L. Chen, Z. Deng, S. Han, Y. Chen, J. Zhong, H. Sun, Y. Lian, B. Yang, X. Yuan, H. Yu, Y. Liu, X. Yang, J. Guo, M. H. Rümmeli, Y. Jiao and Y. Peng, *Advanced Materials*, 2021, **33**, 2101741.
42. E. W. Lees, A. Liu, J. C. Bui, S. Ren, A. Z. Weber and C. P. Berlinguette, *ACS Energy Letters*, 2022, **7**, 1712-1718.
43. W. Luc, X. Fu, J. Shi, J.-J. Lv, M. Jouny, B. H. Ko, Y. Xu, Q. Tu, X. Hu, J. Wu, Q. Yue, Y. Liu, F. Jiao and Y. Kang, *Nature Catalysis*, 2019, **2**, 423-430.
44. J. Li, Y. Kuang, X. Zhang, W.-H. Hung, C.-Y. Chiang, G. Zhu, G. Chen, F. Wang, P. Liang and H. Dai, *Nature Catalysis*, 2023, **6**, 1151-1163.
45. D. Zang, Q. Li, G. Dai, M. Zeng, Y. Huang and Y. Wei, *Applied Catalysis B: Environmental*, 2021, **281**, 119426.
46. X.-F. Qiu, J.-R. Huang, C. Yu, Z.-H. Zhao, H.-L. Zhu, Z. Ke, P.-Q. Liao and X.-M. Chen, *Angewandte Chemie International Edition*, 2022, **61**, e202206470.
47. H. Li, T. Liu, P. Wei, L. Lin, D. Gao, G. Wang and X. Bao, *Angewandte Chemie International Edition*, 2021, **60**, 14329-14333.
48. Y. Ji, Z. Chen, R. Wei, C. Yang, Y. Wang, J. Xu, H. Zhang, A. Guan, J. Chen, T.-K. Sham, J. Luo, Y. Yang, X. Xu and G. Zheng, *Nature Catalysis*, 2022, **5**, 251-258.
49. M. Luo, A. Ozden, Z. Wang, F. Li, J. Erick Huang, S.-F. Hung, Y. Wang, J. Li, D.-H. Nam, Y. C. Li, Y. Xu, R. Lu, S. Zhang, Y. Lum, Y. Ren, L. Fan, F. Wang, H.-h. Li, D. Appadoo, C.-T. Dinh, Y. Liu, B. Chen, J. Wicks, H. Chen, D. Sinton and E. H. Sargent, *Advanced Materials*, 2023, **35**, 2209567.
50. H. Shen, Y. Wang, T. Chakraborty, G. Zhou, C. Wang, X. Fu, Y. Wang, J. Zhang, C. Li, F. Xu, L. Cao, T. Mueller and C. Wang, *ACS Catalysis*, 2022, **12**, 5275-5283.
51. P. Zhu, C. Xia, C.-Y. Liu, K. Jiang, G. Gao, X. Zhang, Y. Xia, Y. Lei, H. N. Alshareef, T. P. Senftle and H. Wang, *Proceedings of the National Academy of Sciences*, 2021, **118**, e2010868118.
52. Y. Liu, S. Chen, X. Quan and H. Yu, *Journal of the American Chemical Society*, 2015, **137**, 11631-11636.
53. Q. Sun, Y. Zhao, X. Tan, C. Jia, Z. Su, Q. Meyer, M. I. Ahmed and C. Zhao, *ACS Catalysis*, 2023, **13**, 5689-5696.
54. F. Ni, H. Yang, Y. Wen, H. Bai, L. Zhang, C. Cui, S. Li, S. He, T. Cheng, B. Zhang and H. Peng, *Science China Materials*, 2020, **63**, 2606-2612.
55. M. Esmaeilirad, A. Kondori, N. Shan, M. T. Saray, S. Sarkar, A. M. Harzandi, C. M. Megaridis, R. Shahbazian-Yassar, L. A. Curtiss, C. U. Segre and M. Asadi, *Applied Catalysis B: Environmental*, 2022, **317**, 121681.
56. H. Xu, D. Rebollar, H. He, L. Chong, Y. Liu, C. Liu, C.-J. Sun, T. Li, J. V. Muntean, R. E. Winans, D.-J. Liu and T. Xu, *Nature Energy*, 2020, **5**, 623-632.
57. Q. Zhang, J. Du, A. He, Z. Liu and C. Tao, *Journal of CO2 Utilization*, 2019, **34**, 635-645.
58. J. Ding, H. Bin Yang, X.-L. Ma, S. Liu, W. Liu, Q. Mao, Y. Huang, J. Li, T. Zhang and B. Liu, *Nature Energy*, 2023, **8**, 1386-1394.

59. X. Wang, Z. Wang, F. P. García de Arquer, C.-T. Dinh, A. Ozden, Y. C. Li, D.-H. Nam, J. Li, Y.-S. Liu, J. Wicks, Z. Chen, M. Chi, B. Chen, Y. Wang, J. Tam, J. Y. Howe, A. Proppe, P. Todorović, F. Li, T.-T. Zhuang, C. M. Gabardo, A. R. Kirmani, C. McCallum, S.-F. Hung, Y. Lum, M. Luo, Y. Min, A. Xu, C. P. O'Brien, B. Stephen, B. Sun, A. H. Ip, L. J. Richter, S. O. Kelley, D. Sinton and E. H. Sargent, *Nature Energy*, 2020, **5**, 478-486.
60. P. Wang, H. Yang, C. Tang, Y. Wu, Y. Zheng, T. Cheng, K. Davey, X. Huang and S.-Z. Qiao, *Nature Communications*, 2022, **13**, 3754.
61. T. T. H. Hoang, S. Ma, J. I. Gold, P. J. A. Kenis and A. A. Gewirth, *ACS Catalysis*, 2017, **7**, 3313-3321.
62. H. Zhang, Y. Qiao, Y. Wang, Y. Zheng and H. Huang, *Sustainable Energy & Fuels*, 2022, **6**, 4860-4865.
63. S. Yan, Z. Chen, Y. Chen, C. Peng, X. Ma, X. Lv, Z. Qiu, Y. Yang, Y. Yang, M. Kuang, X. Xu and G. Zheng, *Journal of the American Chemical Society*, 2023, **145**, 26374-26382.
64. W. Ma, S. Xie, T. Liu, Q. Fan, J. Ye, F. Sun, Z. Jiang, Q. Zhang, J. Cheng and Y. Wang, *Nature Catalysis*, 2020, **3**, 478-487.
65. S. Ma, M. Sadakiyo, R. Luo, M. Heima, M. Yamauchi and P. J. A. Kenis, *Journal of Power Sources*, 2016, **301**, 219-228.
66. C. Reller, R. Krause, E. Volkova, B. Schmid, S. Neubauer, A. Rucki, M. Schuster and G. Schmid, *Advanced Energy Materials*, 2017, **7**, 1602114.
67. C.-T. Dinh, T. Burdyny, M. G. Kibria, A. Seifitokaldani, C. M. Gabardo, F. P. García de Arquer, A. Kiani, J. P. Edwards, P. De Luna, O. S. Bushuyev, C. Zou, R. Quintero-Bermudez, Y. Pang, D. Sinton and E. H. Sargent, *Science*, 2018, **360**, 783-787.
68. J.-J. Lv, M. Jouny, W. Luc, W. Zhu, J.-J. Zhu and F. Jiao, *Advanced Materials*, 2018, **30**, 1803111.
69. T. T. H. Hoang, S. Verma, S. Ma, T. T. Fister, J. Timoshenko, A. I. Frenkel, P. J. A. Kenis and A. A. Gewirth, *Journal of the American Chemical Society*, 2018, **140**, 5791-5797.
70. Y. Wang, Z. Wang, C.-T. Dinh, J. Li, A. Ozden, M. Golam Kibria, A. Seifitokaldani, C.-S. Tan, C. M. Gabardo, M. Luo, H. Zhou, F. Li, Y. Lum, C. McCallum, Y. Xu, M. Liu, A. Proppe, A. Johnston, P. Todorovic, T.-T. Zhuang, D. Sinton, S. O. Kelley and E. H. Sargent, *Nature Catalysis*, 2020, **3**, 98-106.
71. F. P. García de Arquer, C.-T. Dinh, A. Ozden, J. Wicks, C. McCallum, A. R. Kirmani, D.-H. Nam, C. Gabardo, A. Seifitokaldani, X. Wang, Y. C. Li, F. Li, J. Edwards, L. J. Richter, S. J. Thorpe, D. Sinton and E. H. Sargent, *Science*, 2020, **367**, 661-666.
72. A. Inoue, T. Harada, S. Nakanishi and K. Kamiya, *EES Catalysis*, 2023, **1**, 9-16.
73. Z. Li, R. M. Yadav, L. Sun, T. Zhang, J. Zhang, P. M. Ajayan and J. Wu, *Applied Catalysis A: General*, 2020, **606**, 117829.
74. J. Gao, A. Bahmanpour, O. Kröcher, S. M. Zakeeruddin, D. Ren and M. Grätzel, *Nature Chemistry*, 2023, **15**, 705-713.
75. G. O. Larrazábal, V. Okatenko, I. Chorkendorff, R. Buonsanti and B. Seger, *ACS Applied Materials & Interfaces*, 2022, **14**, 7779-7787.

76. W. Liu, P. Zhai, A. Li, B. Wei, K. Si, Y. Wei, X. Wang, G. Zhu, Q. Chen, X. Gu, R. Zhang, W. Zhou and Y. Gong, *Nature Communications*, 2022, **13**, 1877.
77. W. Niu, Z. Chen, W. Guo, W. Mao, Y. Liu, Y. Guo, J. Chen, R. Huang, L. Kang, Y. Ma, Q. Yan, J. Ye, C. Cui, L. Zhang, P. Wang, X. Xu and B. Zhang, *Nature Communications*, 2023, **14**, 4882.
78. K. Qi, Y. Zhang, N. Onofrio, E. Petit, X. Cui, J. Ma, J. Fan, H. Wu, W. Wang, J. Li, J. Liu, Y. Zhang, Y. Wang, G. Jia, J. Wu, L. Lajaunie, C. Salameh and D. Voiry, *Nature Catalysis*, 2023, **6**, 319-331.
79. C. Peng, G. Luo, J. Zhang, M. Chen, Z. Wang, T.-K. Sham, L. Zhang, Y. Li and G. Zheng, *Nature Communications*, 2021, **12**, 1580.
80. X. Wang, P. Ou, A. Ozden, S.-F. Hung, J. Tam, C. M. Gabardo, J. Y. Howe, J. Sisler, K. Bertens, F. P. García de Arquer, R. K. Miao, C. P. O'Brien, Z. Wang, J. Abed, A. S. Rasouli, M. Sun, A. H. Ip, D. Sinton and E. H. Sargent, *Nature Energy*, 2022, **7**, 170-176.
81. Y. Z. Sha Wang, jiajun Zhong, Zhongjun Chen, Yisen Yang,, Buxing Han, Yongxin Cheng, Meiling Li, Qian Li and J. Zhang, *Reserach Square*, 2023, DOI: https://doi.org/10.21203/rs.3.rs-3014897/v1.
82. S. Verma, B. Kim, H.-R. M. Jhong, S. Ma and P. J. A. Kenis, *ChemSusChem*, 2016, **9**, 1972-1979.
83. J. A. Herron and C. T. Maravelias, *Energy Technology*, 2016, **4**, 1369-1391.
84. N. M. Haegel, R. Margolis, T. Buonassisi, D. Feldman, A. Froitzheim, R. Garabedian, M. Green, S. Glunz, H.-M. Henning, B. Holder, I. Kaizuka, B. Kroposki, K. Matsubara, S. Niki, K. Sakurai, R. A. Schindler, W. Tumas, E. R. Weber, G. Wilson, M. Woodhouse and S. Kurtz, *Science*, 2017, **356**, 141-143.
85. O. S. Bushuyev, P. De Luna, C. T. Dinh, L. Tao, G. Saur, J. van de Lagemaat, S. O. Kelley and E. H. Sargent, *Joule*, 2018, **2**, 825-832.
86. M. Jouny, W. Luc and F. Jiao, *Industrial & Engineering Chemistry Research*, 2018, **57**, 2165-2177.
87. J. M. Spurgeon and B. Kumar, *Energy & Environmental Science*, 2018, **11**, 1536-1551.
88. S. M. Jarvis and S. Samsatli, *Renewable and Sustainable Energy Reviews*, 2018, **85**, 46-68.
89. P. De Luna, C. Hahn, D. Higgins, S. A. Jaffer, T. F. Jaramillo and E. H. Sargent, *Science*, 2019, **364**, eaav3506.
90. M. G. Kibria, J. P. Edwards, C. M. Gabardo, C.-T. Dinh, A. Seifitokaldani, D. Sinton and E. H. Sargent, *Advanced Materials*, 2019, **31**, 1807166.
91. S. Verma, S. Lu and P. J. A. Kenis, *Nature Energy*, 2019, **4**, 466-474.
92. E. Pérez-Gallent, S. Turk, R. Latsuzbaia, R. Bhardwaj, A. Anastasopol, F. Sastre-Calabuig, A. C. Garcia, E. Giling and E. Goetheer, *Industrial & Engineering Chemistry Research*, 2019, **58**, 6195-6202.
93. J. Na, B. Seo, J. Kim, C. W. Lee, H. Lee, Y. J. Hwang, B. K. Min, D. K. Lee, H.-S. Oh and U. Lee, *Nature Communications*, 2019, **10**, 5193.

94. M. Ramdin, A. R. T. Morrison, M. de Groen, R. van Haperen, R. de Kler, E. Irtem, A. T. Laitinen, L. J. P. van den Broeke, T. Breugelmans, J. P. M. Trusler, W. d. Jong and T. J. H. Vlugt, *Industrial & Engineering Chemistry Research*, 2019, **58**, 22718-22740.
95. M. Rumayor, A. Dominguez-Ramos, P. Perez and A. Irabien, *Journal of CO2 Utilization*, 2019, **34**, 490-499.
96. S. Y. Chae, S. Y. Lee, S. G. Han, H. Kim, J. Ko, S. Park, O.-S. Joo, D. Kim, Y. Kang, U. Lee, Y. J. Hwang and B. K. Min, *Sustainable Energy & Fuels*, 2020, **4**, 199-212.
97. H. Shin, K. U. Hansen and F. Jiao, *Nature Sustainability*, 2021, **4**, 911-919.
98. S. Overa, T. G. Feric, A.-H. A. Park and F. Jiao, *Joule*, 2021, **5**, 8-13.
99. Á. Vass, B. Endrődi and C. Janáky, *Current Opinion in Electrochemistry*, 2021, **25**, 100621.
100. H. Yadegari, A. Ozden, T. Alkayyali, V. Soni, A. Thevenon, A. Rosas-Hernández, T. Agapie, J. C. Peters, E. H. Sargent and D. Sinton, *ACS Energy Letters*, 2021, **6**, 3538-3544.
101. J. M. Spurgeon, N. Theaker, C. A. Phipps, S. S. Uttarwar and C. A. Grapperhaus, *ACS Sustainable Chemistry & Engineering*, 2022, **10**, 12882-12894.
102. D. Segets, C. Andronescu and U. P. Apfel, *Nat Commun*, 2023, **14**, 7950.
103. I. Bagemihl, L. Cammann, M. Pérez-Fortes, V. van Steijn and J. R. van Ommen, *ACS Sustainable Chemistry & Engineering*, 2023, **11**, 10130-10141.
104. T. Gao, B. Xia, K. Yang, D. Li, T. Shao, S. Chen, Q. Li and J. Duan, *Energy & Fuels*, 2023, **37**, 17997-18008.
105. B. S. Crandall, S. Overa, H. Shin and F. Jiao, *Accounts of Chemical Research*, 2023, **56**, 1505-1516.
106. Y. Li and T.-B. Lu, *Materials Chemistry Frontiers*, 2024, **8**, 341-353.
107. X. Wang, P. Li, J. Tam, J. Y. Howe, C. P. O'Brien, A. Sedighian Rasouli, R. K. Miao, Y. Liu, A. Ozden, K. Xie, J. Wu, D. Sinton and E. H. Sargent, *Nature Sustainability*, 2024, DOI: 10.1038/s41893-024-01363-1.
108. X. Jiang, L. Ke, K. Zhao, X. Yan, H. Wang, X. Cao, Y. Liu, L. Li, Y. Sun, Z. Wang, D. Dang and N. Yan, *Nature Communications*, 2024, **15**, 1427.
